# Supplementary material for: Machine Learning Accelerates Crystallization for Structure Determination
Source: Angew Chem Int Ed Engl. 2026 May 4;65(25):e1218503. doi: 10.1002/anie.1218503 (PMC13266958; doi:10.1002/anie.1218503)

checkCIF/PLATON report

Structure factors have been supplied for datablock(s) lcz039

THIS REPORT IS FOR GUIDANCE ONLY. IF USED AS PART OF A REVIEW PROCEDURE FOR PUBLICATION, IT SHOULD NOT REPLACE THE EXPERTISE OF AN EXPERIENCED CRYSTALLOGRAPHIC REFEREE.

No syntax errors found. CIF dictionary Interpreting this report

Datablock: lcz039

```
Bond precision:   C-C = 0.0101 A          Wavelength=1.54184

Cell:            a=8.2215(2)        b=13.1633(3)      c=16.8689(4)
                 alpha=86.119(2)    beta=83.075(2)    gamma=88.010(2)
Temperature:     100 K
```

	Calculated	Reported
Volume	1807.46(7)	1807.46(7)
Space group	P -1	P -1
Hall group	-P 1	-P 1
Moiety formula	C30 H15 Ag3 F18 N6 O2, H2 O	C15 H3 Ag3 F18 N6, H2 O, C15 H12 O2
Sum formula	C30 H17 Ag3 F18 N6 O3	C30 H17 Ag3 F18 N6 O3
Mr	1175.11	1175.10
Dx,g cm-3	2.159	2.159
Z	2	2
Mu (mm-1)	14.188	14.188
F000	1132.0	1132.0
F000’	1137.37	
h,k,lmax	10,16,21	10,16,21
Nref	7801	7378
Tmin,Tmax	0.125,0.119	0.632,1.000
Tmin’	0.027	

```
Correction method= # Reported T Limits: Tmin=0.632 Tmax=1.000
AbsCorr = MULTI-SCAN
```

Data completeness= 0.946 Theta(max)= 78.712

R(reflections)= 0.0528(6336) wR2(reflections)= 0.1409(7378)

S = 1.070 Npar= 545

The following ALERTS were generated. Each ALERT has the format
test-name_ALERT_alert-type_alert-level.
Click on the hyperlinks for more details of the test.

Alert level C

```
PLAT042_ALERT_1_C Calc. and Reported MoietyFormula Strings  Differ     Please Check
             Calc: C30 H15 Ag3 F18 N6 O2, H2 O
             Rep.: C15 H3 Ag3 F18 N6, H2 O, C15 H12 O2
PLAT213_ALERT_2_C Atom F00C            has ADP max/min Ratio .....        3.1 prolat
PLAT213_ALERT_2_C Atom F00S            has ADP max/min Ratio .....        3.1 prolat
PLAT213_ALERT_2_C Atom F00T            has ADP max/min Ratio .....        3.7 prolat
PLAT213_ALERT_2_C Atom F014            has ADP max/min Ratio .....        3.6 prolat
PLAT234_ALERT_4_C Large Hirshfeld Difference F00R     --C01O     .       0.17 Ang.
PLAT342_ALERT_3_C Low Bond Precision on  C-C Bonds ..............    0.01014 Ang.
PLAT906_ALERT_3_C Large K Value in the Analysis of Variance ......      5.058 Check
PLAT911_ALERT_3_C Missing FCF Refl Between Thmin & STh/L=    0.600        42 Report
               -9  5  0,  -8  7  0,   7 -8  1,   7 -7  1,   8 -6  1,  -9  5  1,
               -8  7  1,  -7  9  1,   4 -8  2,   8 -5  2,  -9  5  2,  -8  7  2,
               -2 15  2,   8 -8  3,   8 -7  3,   8 -5  3,   9 -5  3,   2  0  3,
               -8  7  3,  -7  9  3,   8 -8  4,   8 -7  4,   8 -6  4,   9 -5  4,
                9 -3  4,   8 -8  5,   8 -7  5,   8 -6  5,   8 -5  5,   9 -5  5,
                   (   12 More Missing: see the .ckf listing file)
PLAT971_ALERT_2_C Check Calcd Resid. Dens.  1.11Ang From Ag02          1.80 eA-3
PLAT971_ALERT_2_C Check Calcd Resid. Dens.  1.05Ang From Ag01          1.57 eA-3
PLAT976_ALERT_2_C Check Calcd Resid. Dens.  1.05Ang From N00M    .     -0.65 eA-3
```

Alert level G

```
PLAT007_ALERT_5_G Number of Unrefined Donor-H Atoms .............          3 Report
             H00O  H00A  H00B
PLAT083_ALERT_2_G SHELXL Second Parameter in WGHT  Unusually Large      19.01 Why ?
PLAT154_ALERT_1_G The s.u.'s on the Cell Angles are Equal ..(Note)      0.002 Degree
PLAT242_ALERT_2_G Low    'MainMol' Ueq as Compared to Neighbors of       C01G Check
PLAT242_ALERT_2_G Low    'MainMol' Ueq as Compared to Neighbors of       C01J Check
PLAT242_ALERT_2_G Low    'MainMol' Ueq as Compared to Neighbors of       C01K Check
PLAT242_ALERT_2_G Low    'MainMol' Ueq as Compared to Neighbors of       C01M Check
PLAT242_ALERT_2_G Low    'MainMol' Ueq as Compared to Neighbors of       C01N Check
PLAT242_ALERT_2_G Low    'MainMol' Ueq as Compared to Neighbors of       C01O Check
PLAT720_ALERT_4_G Number of Unusual/Non-Standard Labels ..........         77 Note
             Ag01    Ag02    Ag03    F004    F005    F006    F007    F008
             F009    F00A    O00B    H00A    H00B    F00C    F00D    F00E
             O00F    N00G    F00H    N00I    N00J    F00K    N00L    N00M
             N00N    O00O    H00O    F00P    F00Q    F00R    F00S    F00T
             C00U    H00U    C00V    H00V    C00W    H00W    C00X    H00X
             C00Y    C00Z    H00Z    C010    H010    C011    C012    C013
             H013    F014    C015    C016    C017    C018    H018    C019
             C01A    C01B    H01B    C01C    H01C    C01D    H01D    C01E
```

```
              H01E    C01F    C01G    C01H    H01H    C01I    C01J    C01K
              C01L    H01L    C01M    C01N    C01O
PLAT883_ALERT_1_G Absent Datum for _atom_sites_solution_primary ..     Please Do !
PLAT912_ALERT_4_G Missing # of FCF Reflections Above STh/L=  0.600        381 Note
PLAT913_ALERT_3_G Missing # of Very Strong Reflections in FCF ....          1 Note
                2  0  3,
PLAT933_ALERT_2_G Number of HKL-OMIT Records in Embedded .res File          6 Note
              -10  2  2,  10 -3  1,  -9  5  1,  -9  5  2,  -9 -2  6,  -9  5  0,
PLAT941_ALERT_3_G Average HKL Measurement Multiplicity ..........        2.3 Low
PLAT969_ALERT_5_G The 'Henn et al.' R-Factor-gap value ..........      3.272 Note
              Predicted wR2: Based on SigI**2  4.31 or SHELX Weight 13.17
PLAT978_ALERT_2_G Number C-C Bonds with Positive Residual Density.          0 Info
```

```
   0 ALERT level A = Most likely a serious problem - resolve or explain
   0 ALERT level B = A potentially serious problem, consider carefully
  12 ALERT level C = Check. Ensure it is not caused by an omission or oversight
  17 ALERT level G = General information/check it is not something unexpected

   3 ALERT type 1 CIF construction/syntax error, inconsistent or missing data
  16 ALERT type 2 Indicator that the structure model may be wrong or deficient
   5 ALERT type 3 Indicator that the structure quality may be low
   3 ALERT type 4 Improvement, methodology, query or suggestion
   2 ALERT type 5 Informative message, check
```

Validation response form

Please find below a validation response form (VRF) that can be filled in and pasted into your CIF.

```
# start Validation Reply Form
_vrf_PLAT042_lcz039
;
PROBLEM: Calc. and Reported MoietyFormula Strings  Differ     Please Check
RESPONSE: ...
;
_vrf_PLAT213_lcz039
;
PROBLEM: Atom F00C            has ADP max/min Ratio .....        3.1 prolat
RESPONSE: ...
;
_vrf_PLAT234_lcz039
;
PROBLEM: Large Hirshfeld Difference F00R     --C01O     .       0.17 Ang.
RESPONSE: ...
;
_vrf_PLAT342_lcz039
;
PROBLEM: Low Bond Precision on  C-C Bonds ..............    0.01014 Ang.
RESPONSE: ...
;
_vrf_PLAT906_lcz039
;
PROBLEM: Large K Value in the Analysis of Variance ......      5.058 Check
RESPONSE: ...
;
_vrf_PLAT911_lcz039
```

```
;
PROBLEM: Missing FCF Refl Between Thmin & STh/L=    0.600         42 Report
RESPONSE: ...
;
_vrf_PLAT971_lcz039
;
PROBLEM: Check Calcd Resid. Dens.  1.11Ang From Ag02            1.80 eA-3
RESPONSE: ...
;
_vrf_PLAT976_lcz039
;
PROBLEM: Check Calcd Resid. Dens.  1.05Ang From N00M    .      -0.65 eA-3
RESPONSE: ...
;
# end Validation Reply Form
```

It is advisable to attempt to resolve as many as possible of the alerts in all categories. Often the minor alerts point to easily fixed oversights, errors and omissions in your CIF or refinement strategy, so attention to these fine details can be worthwhile. It is up to the individual to critically assess their own results and, if necessary, seek expert advice.

PLATON version of 17/09/2025; check.def file version of 17/09/2025

---

duplicate check

No duplication found

Datablock lcz039 - ellipsoid plot

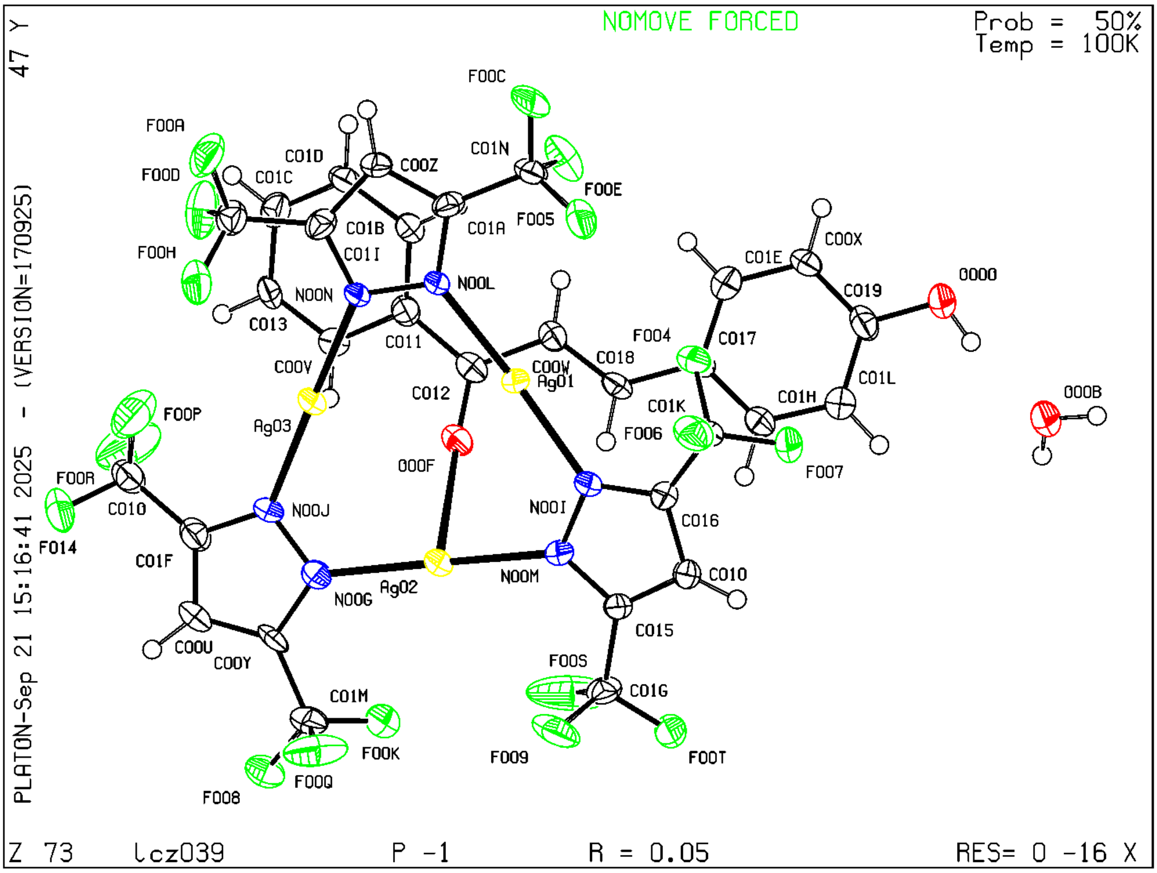

Supplement: Supplementary file 2 — Supporting File 2: anie72462‐sup‐0002‐DataFile.zip. [file ANIE-65-e1218503-s002.zip › supplementary_materials_crystallographic_data submit.zip!/cif_and_checkcif(1-114)/Ag3Pz3_39/Ag3Pz3_39_checkcif.pdf]
